# An asymptomatic perforation of the gastrointestinal tract caused by ingestion of foreign body: A case report

**DOI:** 10.1002/ccr3.8142

**Published:** 2024-01-28

**Authors:** Qiang Wang, Shujie Cheng, Xuejiao Zhang, Tianhao Xie, Xinli Sun, Zheng Niu, Yahan Liu, Xiaoshi Jin

**Affiliations:** ^1^ Department of General Surgery Affiliated Hospital of Hebei University Baoding Hebei Province China; ^2^ Hebei Institute of Dermatology Baoding Hebei Province China

**Keywords:** asymptomatic case report, foreign body, gastrointestinal perforation

## Abstract

Ingestion of foreign bodies is very common in clinical practice. However, gastrointestinal perforation caused by a foreign body is rare, as most foreign bodies can pass the alimentary tract spontaneously or be removed endoscopically. Ingesting a foreign body causes gastrointestinal tract perforation in less than 1% of cases that require surgery. In the past, the literature about gastrointestinal tract perforation caused by foreign bodies had been widely reported worldwide. However, the case of foreign bodies causing gastrointestinal perforation without significant abdominal infection was rarely documented. A 47‐year‐old woman presented with intermittent left lower abdominal pain associated with a mass for 1 month and had no other symptoms. Laparotomy was performed after clinical assessment. During the operation, a local inflammatory mass that adhered to the abdominal wall, part of the small intestine, and sigmoid colon was found in the left lower quarter of the abdominal cavity. The surrounding intestinal wall was edematous. There were two bony foreign bodies in it. Postoperative pathology suggested an inflammatory mass. A foreign body rarely migrates into the abdominal cavity without symptoms that may be related to the omentum's slow perforation process and good function. The best treatment is surgery and using appropriate antibiotics.

## Graphical abstract

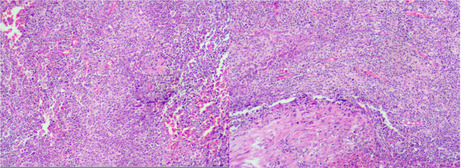

## 1 INTRODUCTION

The ingestion of foreign bodies is very common, but patients rarely can recall the ingestion episode, making it difficult to diagnose, especially when the patient has no particular symptoms.^1^ Foreign objects in the abdomen may be due to involuntary or deliberate input orally or rectally.^2^, ^3^, ^4^ Approximately 80–90% of ingested foreign bodies can pass through the gastrointestinal tract spontaneously without causing symptoms within 1 week. Only less than 1% of the foreign body remained in the lumen, causing severe complications such as intestinal perforation or mucosal injury requiring surgical treatment.^5^ Gastrointestinal perforation is more likely caused by sharp objects over 6.5 cm in length, such as fish bone, chicken bone, and toothpicks^6^

Below, we present a 47‐year‐old woman who arrived with a painful mass in the left lower quadrant of her abdomen and could not recall the history of swallowing any foreign body. During the operation, an inflammatory abundance, including a foreign body suspected as a chicken bone, was found in the left lower enterocele, and there was no perforation or any injury in the gastrointestinal tract surface. There have been no reports of patients in whom the digestive tract spontaneously healed with localized wrapping after a perforation caused by a foreign body. We must consider this possibility in patients with tender masses in clinical practice.

## 2 CASE PRESENTATION

A 47‐year‐old woman presented with a 1‐month history of touching a painful abdominal mass in the left lower quadrant. She denied sexual abuse or sexual accident before and was unaware of having swallowed any foreign bodies. The patient inserted an intrauterine contraceptive device 10 years ago and took it out without complications. She did not report fever, chills, nausea, vomiting, melena, weight loss, rectal bleeding, appetite change, recent illness, or infectious contacts. She did not have any abdominal surgeries previously. There was pain without rebound tenderness when we touched her left lower quadrant of the abdominal wall. Based on the patient's history and physical examination, the initial diagnosis we considered was an abdominal tumor.

**FIGURE 1:**
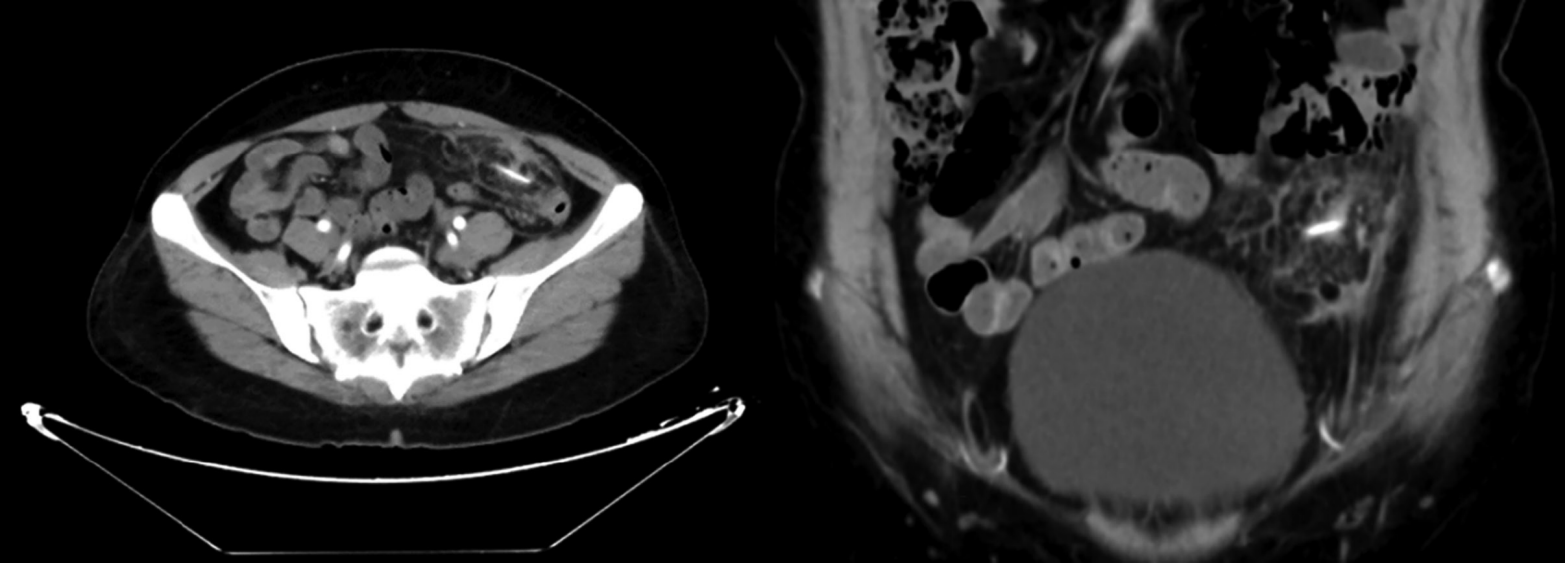
CT scan of the abdominal mass and high‐density lesion.

**FIGURE 2:**
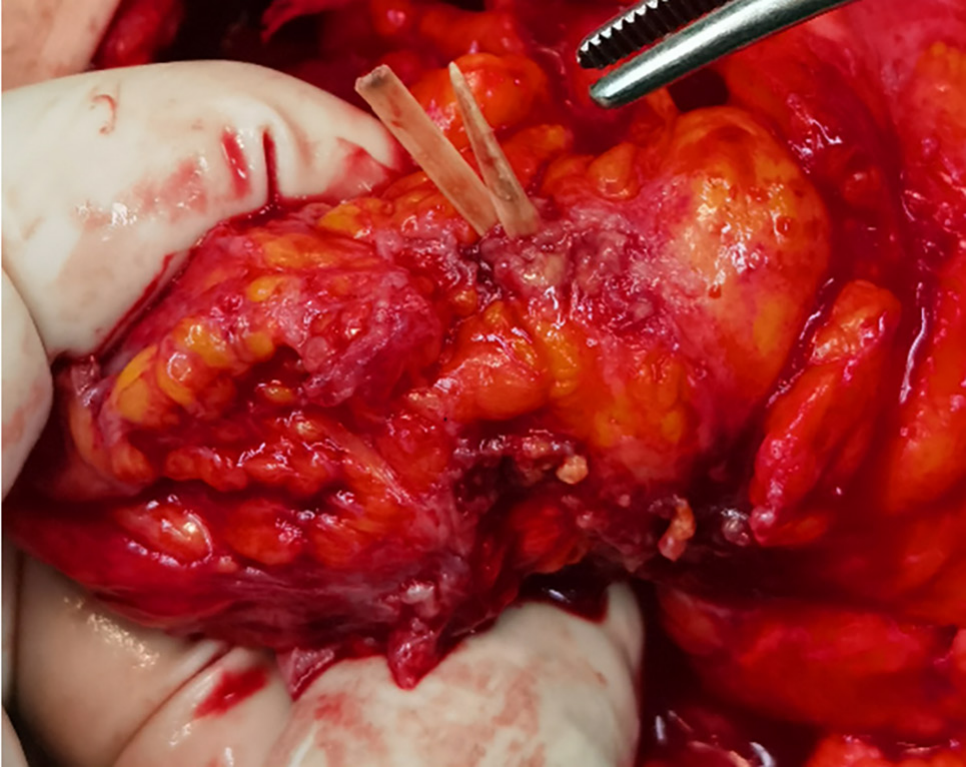
Bone‐like foreign body punctured from the mass.

**FIGURE 3:**
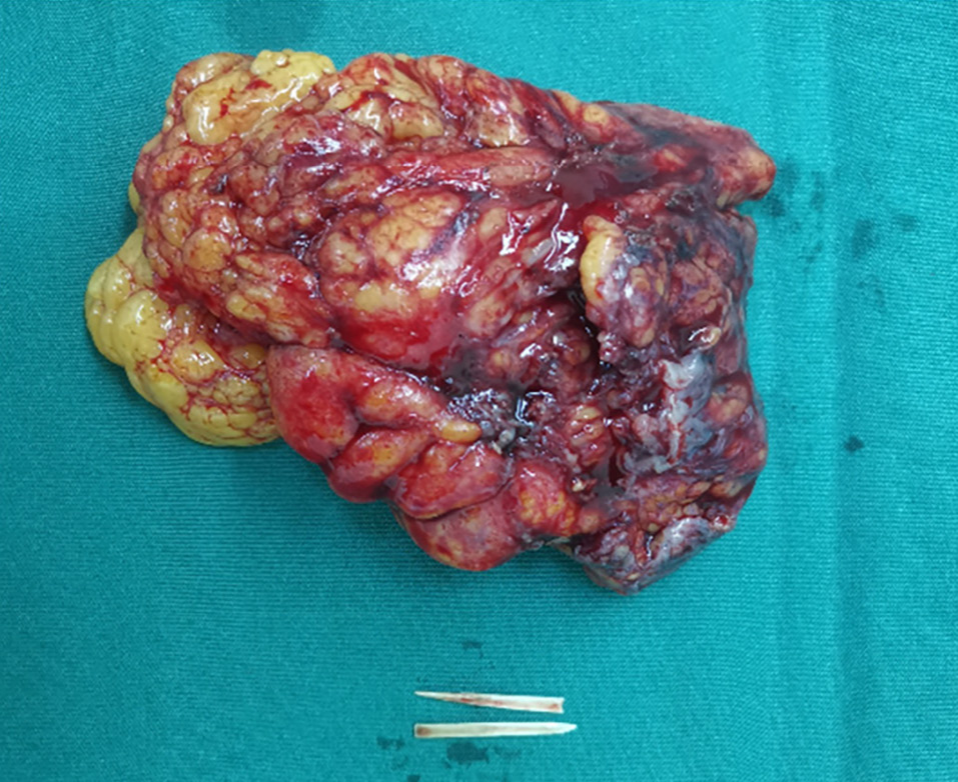
Inflammatory mass and foreign body.

**FIGURE 4:**
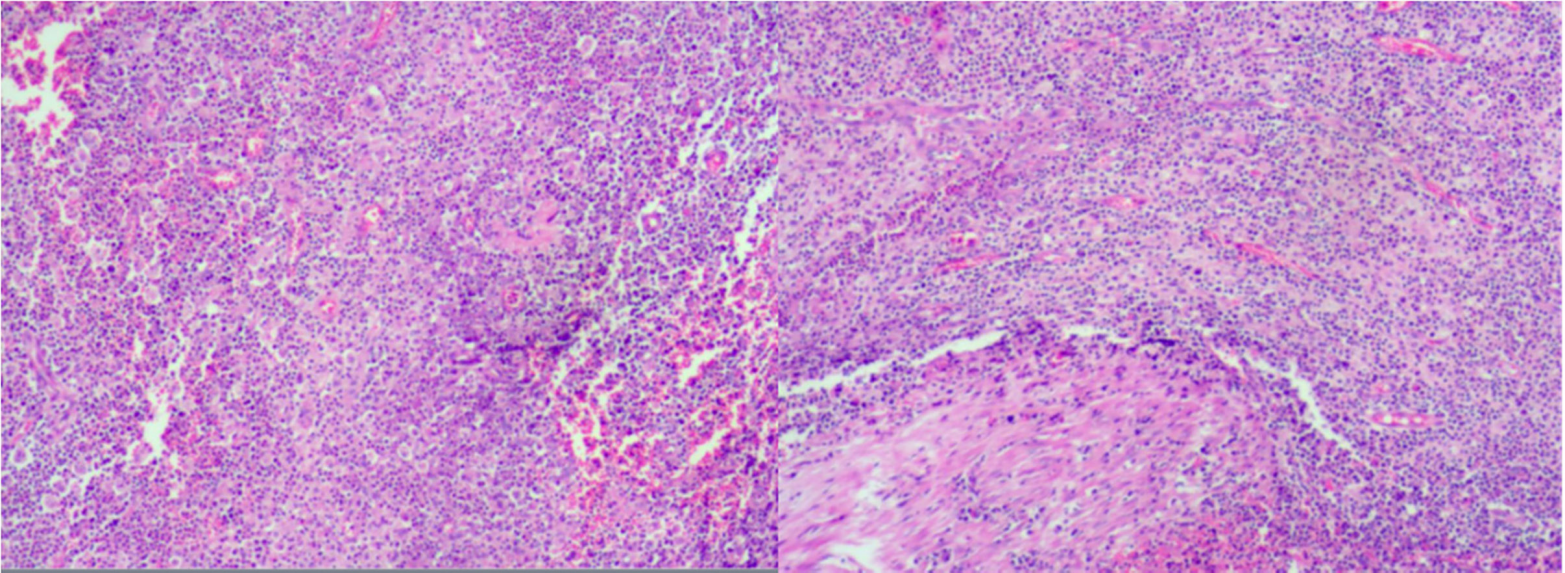
Inflammatory purulent lesion with central necrosis containing pus and actinomycotic cells.

Her basic blood biochemistry revealed that the white blood cell count was 5.66 × 10^9^/L, the C‐reactive protein concentration was lower than 0.5 mg/L, and the tumor marks were average. The CT scan revealed a large, irregular‐shaped, mimicking honeycomb heterogeneous mass with posterior enhancement, and there was a high‐density lesion in the center (Figure 1). The investigation of colonoscopy was routine, and an exploratory laparotomy was conducted. There was no perforation in the GI tract. A tumor‐like inflammatory mass enwrapped by omentum and adhered to the anterior peritoneum, the sigmoid colon, and part of the jejunum was revealed in the left lower of enterococci (Figure 2). The involved intestinal wall was edematous. After releasing the adhesion around the tumor, a bone‐like foreign body punctured from the inflammatory mass; then, we performed an excision with the inflammatory mass and partial resection of the omentum (Figure 3). The histopathological examination showed an inflammatory purulent lesion with central necrosis containing pus and actinomycotic cells (Figure 4). The patient received parenteral crystalline penicillin treatment and fully recovered without any complications. We take a follow‐up 2 months later. The patient had a good diet without chills, fever, and abdominal pain; her fart and defecation were average.

## 3 DISCUSSION

Accidental ingestion of foreign bodies was widespread, especially in children and adults with psychological problems.^1^, ^7^ Most foreign bodies were ingested orally, with 80–90% of ingested foreign bodies spontaneously passing through the gastrointestinal tract uneventfully within 1 week,^5^ and some of that was involuntary or deliberate input rectally^4^; in addition, they also could be the result of iatrogenic complications during surgical and interventions procedures, such as intrauterine device and surgical sponge and so on.^8^, ^9^ Less than 1% of patients who ingested a foreign body became symptomatic, caused by perforation of the gastrointestinal tract and required surgery.^1^, ^7^, ^10^, ^11^ The longer, stiffer, and more pointed foreign bodies that included fish or chicken bone, toothpick, pen, and jujube pit were more likely to cause gastrointestinal perforation. The most common location where intestinal perforation occurs following ingestion of a foreign body is the points of physiological angulation or narrowing within the digestive tract, such as the duodenal loop, duodenojejunal junction, terminal ileum, sigmoid colon, and appendix. In the present study, the patients whose digestive tract perforation was caused by a foreign body had varied clinical presentations, including acute peritonitis, intra‐abdominal abscess, liver abscess, epidural abscess, abdominal wall actinomycosis, peritoneal granulomas, or completely asymptomatic. Gastrointestinal injuries caused by foreign bodies are typically diagnosed by CT scan and endoscopy. There is no doubt that patients who suffer acute peritonitis need surgery. According to previous literature, endoscopy, laparoscopy, or laparotomy is suggested after clinical assessment when patients do not present with acute peritonitis but have other symptoms, such as localized tenderness.^12^, ^13^

In this patient, she only had a painful abdominal mass in the left lower quadrant and no other symptoms, which may be caused by a slow perforation of foreign bodies in the thicker gastrointestinal tract, which includes the stomach, duodenum, and large intestine.^7^ If the process is slow enough, the omentum will wrap foreign bodies, and the intestinal damage will heal gradually. This course may also explain why she has no intestinal damage in her colonoscopy and laparotomy. She could not recall the memory of any foreign bodies. Similar cases have been reported in previous literature.^10^ Asymptomatic gastrointestinal perforation is rare in normal adults; most are children or older people with intellectual disabilities. In addition, the cause of the inability to recall the experience of swallowing foreign bodies may be related to the delayed perforation of foreign bodies. The foreign bodies may remain dormant for months or even years until presentation and calcifications indicate chronicity, as mentioned in a case report.^14^ It was difficult for clinicians to diagnose and treat patients in time. Even though some patients may be asymptomatic or have non‐specific symptoms, and most patients refuse to give an accurate history, a detailed interrogation of history is still necessary.

In addition, there is a need for CT scans and endoscopy. The most sensitive and specific examination to diagnose the presence and location of intestinal perforation is a CT scan.^15^ The endoscope can not only locate the perforation site but also observe the digestive tract damage area of the foreign body more visually and remove the foreign object in some patients.^13^ Although laparoscopic surgery is preferred to reduce the patient's trauma, laparotomy is also worth considering.^12^ We chose laparotomy due to the possibility of severe adhesions in the patient's abdominal cavity, which could lead to gastrointestinal damage during abdominocentesis, and to prevent the spread of abdominal infection. The procedure took less time and was performed in our original peritoneal drying method, which reduced the risk of residual abdominal infection and accelerated the recovery of gastrointestinal function.

## 4 CONCLUSION

A foreign body rarely migrates into the abdominal cavity without symptoms, which may be related to the foreign body's slow perforation process and the good function of the omentum. In our experience, CT scans play an essential role in diagnosis; the best treatment is surgery using appropriate antibiotics.

## AUTHOR CONTRIBUTIONS

**Qiang Wang:** Writing – original draft. **Shujie Cheng:** Project administration. **Xuejiao Zhang:** Funding acquisition. **Tianhao Xie:** Conceptualization; investigation. **Xinli Sun:** Resources. **Zheng Niu:** Resources. **Yahan Liu:** Resources. **Xiaoshi Jin:** Writing – review and editing.

## FUNDING INFORMATION

None.

## CONFLICT OF INTEREST STATEMENT

The authors declare that they have no competing interests.

## ETHICS STATEMENT

None.

## CONSENT

Written informed consent was obtained from the patient for publication of this case report and any accompanying images. A copy of the written consent is available for review by the Editor of this journal.

## Data Availability

Data sharing is not applicable to this article as no new data were created or analyzed in this study.
